# Diversity and persistence of MRSA and VRE in nursing homes: Environmental screening and whole-genome sequencing

**DOI:** 10.1017/ash.2022.208

**Published:** 2022-05-16

**Authors:** Marco Cassone, Joyce Wang, Bonnie Lansing, Julia Mantey, Kristen Gibson, Kyle Gontjes, Lona Mody

## Abstract

**Background:** Transmission of methicillin-resistant *Staphylococcus aureus* (MRSA) and vancomycin-resistant *Enterococcus* (VRE) is of special concern among frail patients in nursing homes. To understand environmental contamination patterns in this setting, we screened a suitable section of a nursing home over time and assessed MRSA and VRE prevalence in patients and their rooms. We were especially interested in assessing whether MRSA and VRE strains persist in rooms during changes of occupancy after patient discharge. **Methods:** We conducted a prospective cohort study of MRSA and VRE colonization and contamination among successive patients in a cluster of 9 single-occupancy rooms. Using flocked swabs, 5 high-touch surfaces were screened 3 times a week for 34 weeks. Patients were also screened (ie, nares, groin, and hands), if they agreed to participate. Whole-genome sequencing was performed on 67 nonredundant MRSA and VRE strains. Single-nucleotide polymorphism heatmaps and similarity trees were generated to evaluate strain diversity and persistence the facility. **Results:** Overall, 146 distinct occupancy events were captured during the study (16.5 average per room; range, 11–22), with 387 study visits and 4,670 total swabs collected. All rooms were contaminated with VRE, and 8 of 9 were contaminated with MRSA at least once during the study period. New contamination of a room with MRSA or VRE was observed in 43 (23%) of 185 opportunities, with potential persistence during occupancy changes in 25 (32.9%) of 76 opportunities. Sequencing of 67 nonredundant isolates identified at least 6 enterococcal clades and 10 MRSA clades (6 USA100 and 4 USA300), indicating a high degree of diversity and probably multiple introductions in the facility during the study time. In 3 separate cases, whole-genome sequencing confirmed persistence of a specific MRSA strain during a change of room occupancy, including 1 case of a MRSA strain persisting in a clean room before admission of the next patient. For VRE, 2 cases of persistence during room occupancy changes were confirmed, along with 6 cases of possible persistence (contamination across noncontiguous room occupancy events). **Conclusions:** Active surveillance screening and a recurring evaluation of terminal cleaning procedures should be considered due to high levels of circulation and persistence of MRSA and VRE in the nursing home setting.

**Funding:** None

**Disclosures:** None

